# Effects of the unity vacuum suspension system on transtibial gait for simulated non-level surfaces

**DOI:** 10.1371/journal.pone.0199181

**Published:** 2018-06-14

**Authors:** Gabrielle Thibault, Hossein Gholizadeh, Emily Sinitski, Natalie Baddour, Edward D. Lemaire

**Affiliations:** 1 The Ottawa Hospital Rehabilitation Centre, Centre for Rehabilitation Research and Development, Ottawa, Canada; 2 Canadian Forces Health Services, Ottawa, Canada; 3 Faculty of Engineering, Department of Mechanical Engineering, University of Ottawa, Ottawa, Canada; 4 Faculty of Medicine, University of Ottawa, Ottawa, Canada; Nanyang Technological University, SINGAPORE

## Abstract

Walking on various surfaces encountered in everyday life requires lower limb prosthesis users to continually adapt their movement patterns. Elevated vacuum suspension systems could improve transtibial amputee gait on non-level surfaces; however, research is lacking to guide clinical practice. Twelve transtibial amputees were fitted with the Össur sleeveless vacuum suspension system (Unity). After a one month accommodation period, the CAREN-Extended system was used to evaluate gait on a self-paced treadmill when walking with continuous perturbations (medial-lateral translations, rolling hills, simulated uneven ground) with an active or inactive vacuum suspension system. Significant differences between active and inactive vacuum conditions (p<0.05) were found for some temporal-spatial and kinematic gait parameters, but the differences were small and not considered clinically significant. Our findings suggest that potential vacuum pump failures would not immediately affect gait performance in a moderately high functioning amputee population. However, residual limb volume changes over time due to the removal of elevated vacuum may adversely affect socket fit, leading to greater gait differences and reduced quality of life.

## Introduction

A prosthetic socket is the critical component that connects the residual limb to the lower limb prosthesis. This interface is essential for comfort, gait symmetry, proprioception, and amputee satisfaction [1–4].Therefore, selecting an appropriate suspension system is an important step in prosthetic rehabilitation to ensure the prosthesis is attached securely and efficiently to the residual limb.

Based on the literature [2,5–7], vacuum assisted suspension system (VASS) could improve prosthetic function, comfort, satisfaction, and quality of life compared to other prosthetic suspension systems on the market such as single distal pin/lock, lanyard, suction, or Kondylen Bettung Munster (KBM) suspension system. Board et al. demonstrated that step length and stance time were more asymmetric when walking with inactive vacuum (suction suspension) compared to active vacuum suspension [2]. Ferreira and Neves also reported that level walking asymmetry decreased with elevated vacuum [8]. Nevertheless, VASS may not be appropriate for all amputees because more attention and skills are needed for donning the liner, socket, and sleeve. Moreover, an external sleeve (i.e., suspension and vacuum seal layer) that covers the socket, knee, and lower thigh can limit knee range of motion [5]. The Unity elevated vacuum suspension system (Össur) was developed to address these limitations.

The Unity system does not require an external knee sleeve and consists of a mechanical vacuum pump and a hypobaric sealing membrane around a silicon liner, so no external sleeve or shuttle lock is required. Seal-In V is available with the seal in two different areas; standard and high profile. The high profile Seal-In V liner can be used in case of sensitive spots on the distal tibial crest or for longer residual limbs [9]. Based on the manufacturer guidelines [9], proper stump length is 10–13 cm for the standard profile and 13–16 cm for the high profile liner. Seal-In V seal height is fixed at 5 mm in standard profile and 35 mm from the start of the distal radius in high profile. Negative pressure (vacuum) is created below the seal area in this suspension system, while in other systems with a sleeve apply vacuum to the whole residual limb. Therefore, the vacuum effects on the residual limb may not be the same between Unity and other vacuum systems. The earliest elevated vacuum study was published by Board et al. in 2001, where active vacuum decreased stump volume changes and pistoning compared to inactive vacuum (suction) [2]. Moreover, step length and stance time were more symmetric with active vacuum. Ferreira & Neves [8] reported that the elevated vacuum systems improved gait symmetry between the prosthetic and intact limbs, compared to the Kondylen Bettung Munster (KBM) suspension system. However, no statistically significant differences were found for the temporal-spatial parameters. Xu et al. (2017) also applied vacuum levels of 0, 5, 10, 15, and 20 inHg to explore vacuum level effects on gait characteristics for unilateral transtibial amputees. They reported no statistically significant effects on temporal-spatial parameters [10].

In everyday life, people must continually adapt their movement patterns as they walk over continuously variable terrain. These conditions are more challenging than level walking for amputees due to the increased biomechanical demands on non-level surfaces [11,12]. When walking on uneven ground, able-bodied and people with lower limb amputation adopt a more cautious gait pattern, characterized by slower walking velocity with wider step width and shorter step length, compared to walking on level surfaces [12–14]. Moreover, ankle, knee, and hip flexion increase at initial contact, contributing to an overall lowering of the body over non-level surfaces to increase stability while walking [11–13,15].

To date, gait with different vacuum suspension systems have only been studied on level ground. Although gait differences between active and inactive vacuum conditions were small over level ground [5,8,10,16], elevated vacuum suspension has shown to improve proprioception and fit [1,2,5,6,7,8], which could have a considerable impact on gait for more challenging walking surfaces. Therefore, research is needed to better understand walking performance for individuals with a transtibial amputation when walking with the Unity system on non-level surfaces.

The goal of this research is to compare transtibial amputee gait when using the Unity suspension system with inactive vacuum (suction system) and active elevated vacuum suspension, when walking on community-relevant surfaces with continuous perturbations. Temporal-spatial (e.g., step length and step time) and lower limb kinematics (e.g., ankle, knee, and hip angles) were evaluated when walking with active and inactive Unity suspension system. We hypothesized that temporal-spatial and kinematic gait parameters would be different between elevated vacuum and inactive vacuum (i.e., suction socket suspension) conditions. The research outcomes will help to guide VASS prescription for prosthetic rehabilitation by better understanding the direct effects on walking when using this technology.

## Materials and methods

A convenience sample of twelve people with transtibial amputation were recruited through The Ottawa Hospital Rehabilitation Centre (TOHRC), Prosthetics and Orthotics Service (Table 1). Recruiting subjects for this study started in 2016. Participants included in this study were individuals with unilateral transtibial amputation who could walk without walking aids, had steady limb volume during the previous year, and used prosthesis daily. Participants had no gait or balance problems, based on observing their gait during an evaluation session. Participants were excluded if they reported joint pain, stroke, visual impairment, cognitive problems that adversely affect gait and balance, or residual limb length less than 10 cm. Based on manufacturer guidelines, 10 cm is the minimal residual length for the Standard Iceross Seal-In V liner [9]. Medications were also reviewed for side effects related to balance and gait. Individuals with a recent amputation (< 1 year) were also excluded from this study since significant changes in residual limb volume observed during the first year can affect comfort and gait performance [17] as well as require frequent socket adjustments. In this study, time since amputation was on average 13 years (2–74 years). Participants required approximately seven (SD = 3) sessions for casting, socket adjustment, alignment, gait training, and troubleshooting before participating in the walking analysis trials. Ten participants were K-level 3 (i.e., ability or potential for ambulation with variable cadence) and two participants were K-level 4 (i.e., ability or potential for prosthetic ambulation that exceeds basic ambulation skills, exhibiting high impact, stress, or energy levels) [18]. The study protocol was approved by The Ottawa Hospital Research Ethics Board.

**Table 1:** Participant characteristics.

Sex	Age (year)	Height (cm)	Weight (kg)	Amputation (years)	Amputation cause	Stump circumference (cm)^¶^	Stump length (cm)^×^	Activity level	Previous suspension	Gait training sessions
F^¤^	44	164	81	22	Trauma	28.0	13.0	K3	Pin/lock	6
M»	85	175	97	2	Infection	31.0	15.0	K3	Pin/lock	7
M	29	173	72	5	Trauma	26.0	16.0	K3	Suction	5
M	53	186	85	12	Trauma	27.0	18.0	K4	Suction	4
M	52	180	86	13	Diabetic	25.5	13.5	K4	Pin/lock	6
M	70	179	77	9	Diabetic	25.0	13.0	K3	Pin/lock	7
M	48	185	93	3	Trauma	29.5	18.0	K3	Pin/lock	5
M	61	175	94	2	Trauma	32.0	18.0	K3	Suction	10
M	76	178	81	74	Trauma	23.7	12.5	K3	Pin/lock	15
M	63	175	83	6	Trauma	25.0	15.0	K3	Pin/lock	8
M	59	185	134	4	Diabetic	35.0	20.0	K3	Pin/lock	10
M	46	184	104	5	Trauma	28.0	23.0	K3	Pin/lock	6
Mean	57.2	178.3	90.6	13.1		28	16.2			**7**
SD	15.3	6.4	16.4	20.0		3.38	3.2			3

^¶^Stump circumference was measured 4cm from the residual limb’s distal end (circumferential measure) and

^×^stump length was measured from the inferior edge of the patella to distal end of the stump.

^»^M = Male,

^¤^F = Female.

### Prosthetic fabrication and training

According to manufacturer guidelines [9], a Pro-Flex XC foot with Unity pump and Iceross Seal-In V liner were used to fabricate a transtibial prosthesis for each participant. To ensure consistent prosthetic procedures throughout the study and reduced bias, one prosthetist completed all casting, modification, socket fabrication, and alignment. Gait training sessions were provided for all participants, including walking on level surface, stairs, slopes, and uneven ground. The number of training sessions differed for each participant (Table 1). During prosthetic training sessions, a vacuum gauge was used to check negative pressure inside the socket. Negative pressures between -16 and -20 inHg were achieved for all participants. Following prosthetic training, participants wore the prosthesis for a one month acclimation period.

### Data collection

**Fig 1:**
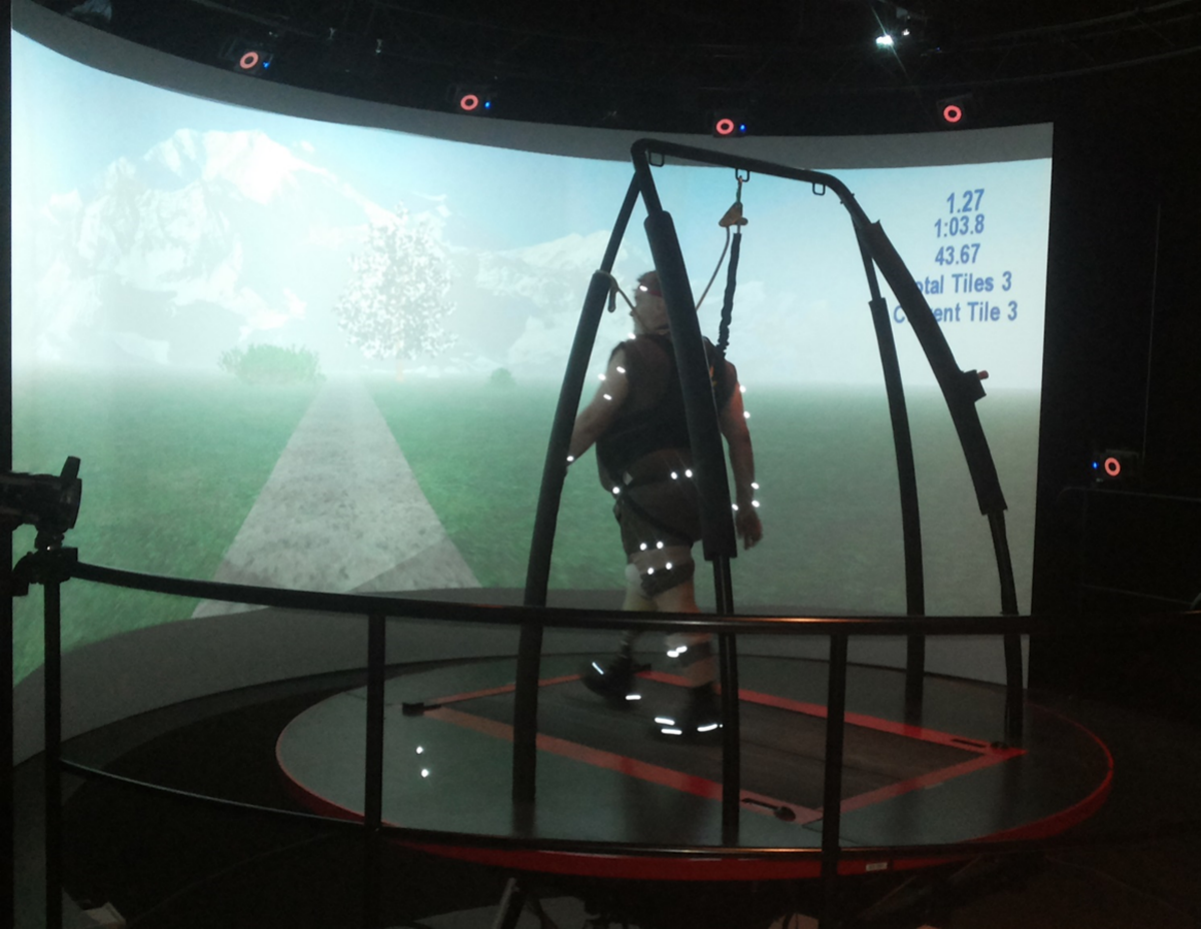
Participant walking in the CAREN-Extended with Park virtual environment.

The CAREN-Extended virtual reality system (Motek Force Link, Amsterdam, NL) was used in this study. CAREN-Extended combines a six degree-of-freedom motion platform with embedded dual-belt instrumented treadmill [19], 180 degree screen to display a 3D virtual Park scenario, 12 camera Vicon motion capture system, and safety harness frame (Fig 1). Fifty-seven markers [20] were used to track full body kinematics and platform motion was tracked by three reflective markers. Virtual markers (digitizing landmarks) were used to define segment ends (S1 Appendix). Kinematic data were collected at 100 Hz.

To become familiar with treadmill self-paced mode and the Park virtual scenario, all participants completed a 10-min warm-up trial. Following the warm-up trial, participants completed two walking trials (340 meter) while the vacuum was inactive (OFF) and two walking trials while the vacuum was active (ON). For the active condition, the distal tube remained correctly connected to the foot. The tube was detached for the inactive condition. For the active condition, the distal Unity tube remained correctly connected to the foot. The tube was detached for the inactive condition and the one-way valve was pushed to remove negative pressure inside the socket. Presentation order for ON or OFF condition were randomized and blinded. Walking trials consisted of level, 7° uphill, -7° downhill, 5° side slopes, medial-lateral translations (ML), rolling hills (HL), and simulated uneven ground (RO). Participants were provided rest breaks between trials to minimize fatigue. For the purposes of this paper, only walking at self-selected speed with medial-lateral translations, rolling hills, and simulated uneven ground were analyzed. During medial-lateral (ML) translations, the platform oscillates in the medial-lateral direction based on a sum of four sines with frequencies of 0.16, 0.21, 0.24, and 0.49 Hz. Amplitude scaling, Aw = 0.015, yields a maximum range of ±4 cm. For rolling-hills (HL), the platform oscillates in the sagittal plane (pitch) based on a sum of four sines with frequencies of 0.16, 0.21, 0.24, and 0.49 Hz. Amplitude scaling, Aw = 0.01, yields a maximum range of ±3°. Rocky (RO) condition involved platform oscillations in three directions simultaneously using the CAREN Rumble module with a maximum range of ±2 cm at 0.6 Hz vertically, ±1° at 1 Hz pitch, and ±1° at 1.2 Hz roll [21].

### Data analysis

A 4th order low-pass Butterworth filter with a 10 Hz cut-off frequency was used to filter marker data. Vicon Nexus software (version 2.3, UK) was used to label the markers. Visual3D (version 6.00.31, USA) was used to generate a 13 segment model (feet, shank, thigh, pelvis, trunk, head, upper arms, and forearms), identify gait events, and calculate joint kinematics. Ten gait cycles were selected for each condition (ON and OFF). Data were time normalized to 0–100% gait cycle. SPSS software (version 23.0, USA) was used for statistical analysis. Two-tailed Wilcoxon signed-rank tests were performed to examine temporal-spatial and gait parameter differences for prosthetic and intact limb separately with vacuum ON or OFF.P-values were adjusted using the Holm-Bonferroni method [22] to control for the likelihood of type I error. Effect size (d) was also calculated to determine the standardised difference between two means, with d<0.2 considered very small effect size, 0.5>d = >0.2 a small effect size, 0.5< d <0.8 a medium effect size, and d <0.8 considered large effect size. Limb symmetry was calculated using a Symmetry Index (SI) (Eq 1). Based on Åström and Stenström, a SI less than 10% shows good symmetry [23]:

$$SI=\frac{Sound\;limb\;value-Prosthetic\;limb\;value}{\frac{1}{2}\left(Sound\;limb\;value+Prosthetic\;limb\;value\right)}*100$$

Descriptive statistics were used to compare non-level surfaces, to provide a basis for understanding the general differences between these walking scenarios for transtibial amputees.

## Results

### Temporal-spatial gait parameters

Means and standard deviations for temporal-spatial gait parameters are reported in Table 2. No significant differences were found between the vacuum suspension system (ON) and the suction suspension system (OFF) when walking with medial-lateral translations, for all temporal-spatial gait parameters evaluated. Significant differences were found between stride length (p = 0.026), swing time (p = 0.034), and step width (p = 0.005) during rolling hills for the prosthetic limb; however the effect sizes were small (d<0.5).

**Table 2:** Mean and standard deviation (in brackets) of temporal-spatial gait parameters with the elevated vacuum system ON and OFF for medial-lateral (ML), rolling hills (HL), and rocky (RO) surfaces.

Medial-Lateral								
	Prosthetic Limb				Intact limb			
	OFF	ON	*p*^≠^	*d*^≠^	OFF	ON	*p*	*d*
Velocity (m/s)	1.13 (0.24)	1.13 (0.20)	0.762	0.1	1.16 (0.25)	1.14 (0.20)	0.169	0.2
Stride Length (m)	1.25 (0.20)	1.26 (0.16)	0.745	0.1	1.28 (0.20)	1.28 (0.16)	0.637	0.1
Step Length (cm)	61.03 (10.61)	60.98 (7.82)	0.656	0.1	65.27 (10.32)	65.72 (8.33)	0.939	0.1
Step Width (cm)	14.70 (6.71)	14.33 (6.44)	0.738	0.1	14.44 (5.35)	13.87 (4.30)	0.242	0.2
Stride Time (s)	1.13 (0.11)	1.13 (0.11)	0.902	0.0	1.12 (0.12)	1.13 (0.11)	0.063	0.3
Step Time (s)	0.57 (0.06)	0.57 (0.06)	0.626	0.1	0.56 (0.06)	0.57 (0.05)	0.204	0.2
Stance Time (s)	0.73 (0.08)	0.73 (0.08)	0.746	0.1	0.75 (0.10)	0.75 (0.09)	0.152	0.2
Swing Time (s)	0.40 (0.04)	0.40 (0.04)	0.785	0.0	0.38 (0.02)	0.39 (0.03)	0.328	0.2
DST (s)^¶^	0.17 (0.03)	0.17 (0.03)	0.664	0.1	0.18 (0.04)	0.18 (0.04)	0.731	0.1
SLT (s)^¤^	0.38 (0.04)	0.39 (0.03)	0.618	0.1	0.40 (0.04)	0.41 (0.03)	0.084	0.3
Symmetry Index (SI)								
	OFF				ON			
SI-Step Length (%)	11.2 (9.6)				12.2 (9.1)			
SI-Step Time (%)	6.3 (5.0)				5.9 (4.6)			
SI-Stance Time (%)	5.4 (5.6)				5.3 (4.3)			
Rolling hills								
	Prosthetic Limb				Intact limb			
	OFF	ON	*p*	*d*	OFF	ON	*p*	*d*
Velocity (m/s)	1.00 (0.30)	0.97 (0.22)	0.194	0.2	1.02 (0.30)	0.98 (0.23)	0.175	0.2
Stride Length (m)	1.13 (0.26)	1.11 (0.20)	0.026*	0.3	1.15 (0.27)	1.12 (0.21)	0.270	0.2
Step Length (cm)	54.45 (12.06)	54.24 (10.56)	0.992	0.0	57.49 (14.05)	59.89 (10.69)	0.185	0.2
Step Width (cm)	15.03 (7.69)	13.55 (7.27)	0.005*	0.4	14.99 (4.11)	14.15 (3.93)	0.010*	0.4
Stride Time (s)	1.15 (0.11)	1.16 (0.12)	0.625	0.1	1.16 (0.11)	1.16 (0.12)	0.464	0.1
Step Time (s)	0.58 (0.06)	0.58 (0.07)	0.346	0.1	0.58 (0.06)	0.58 (0.06)	0.641	0.1
Stance Time (s)	0.76 (0.10)	0.77 (0.10)	0.333	0.1	0.79 (0.10)	0.79 (0.11)	0.698	0.1
Swing Time (s)	0.39 (0.03)	0.40 (0.04)	0.034*	0.3	0.37 (0.03)	0.37 (0.03)	0.932	0.0
DST (s)	0.19 (0.05)	0.18 (0.04)	0.291	0.2	0.21 (0.05)	0.21 (0.05)	0.932	0.0
SLT (s)	0.36 (0.05)	0.38 (0.05)	0.291	0.1	0.40 (0.00)	0.40 (0.04)	0.152	0.2
Symmetry Index (SI)								
	OFF				ON			
SI-Step Length (%)	13.6 (9.3)				14.8 (11.1)			
SI-Step Time (%)	7.0 (5.8)				7.3 (5.1)			
SI-Stance Time (%)	6.4 (4.3)				6.1 (4.7)			
Rocky								
	Prosthetic Limb				Intact limb			
	OFF	ON	*p*	*d*	OFF	ON	*p*	*d*
Velocity (m/s)	1.07 (0.28)	1.06 (0.25)	0.110	0.2	1.08 (0.29)	1.07 (0.25)	0.067	0.3
Stride Length (m)	1.19 (0.26)	1.19 (0.25)	0.252	0.1	1.19 (0.27)	1.21 (0.25)	0.863	0.0
Step Length (cm)	57.57 (12.54)	57.68 (12.29)	0.160	0.1	61.41 (14.10)	63.27 (13.60)	0.839	0.0
Step Width (cm)	14.43 (6.91)	15.03 (7.00)	0.028*	0.4	14.80 (3.77)	16.00 (5.18)	0.009*	0.5
Stride Time (s)	1.13 (0.12)	1.14 (0.11)	0.330	0.0	1.12 (0.13)	1.14 (0.11)	0.011*	0.4
Step Time (s)	0.56 (0.07)	0.56 (0.06)	0.584	0.2	0.56 (0.06)	0.58 (0.05)	0.005*	0.5
Stance Time (s)	0.73 (0.09)	0.74 (0.08)	0.086	0.2	0.74 (0.10)	0.75 (0.08)	0.019*	0.4
Swing Time (s)	0.40 (0.04)	0.40 (0.05)	0.718	0.0	0.38 (0.04)	0.39 (0.04)	0.125	0.2
DST (s)	0.17 (0.04)	0.17 (0.04)	0.187	0.1	0.18 (0.03)	0.19 (0.04)	0.020*	0.4
SLT (s)	0.39 (0.05)	0.40 (0.04)	0.450	0.1	0.41 (0.05)	0.40 (0.04)	0.215	0.2
Symmetry Index (SI)								
	OFF				ON			
SI-Step Length (%)	13.4 (11.2)				14.0 (9.1)			
SI-Step Time (%)	6.5 (5.7)				6.7 (6.6)			
SI-Stance Time (%)	5.4 (5.0)				5.5 (3.9)			

^¶^DST = double support time,

^¤^SLT = single limb support, SI = symmetry index.

^≠^*p* and *d* are between OFF and ON results.

* *p*<0.05.

Results showed a significant difference (p = 0.028) between ON and OFF conditions for step width on the rocky surface for the prosthetic limb; however, the difference was less than 0.7cm.

Good symmetry (SI<10%) was found between prosthetic and intact limbs for step time and stance time with both ON and OFF conditions during gait with continuous perturbations. However, step length was not symmetrical (SI>10%) for all walking surfaces.

Participants increased step length and walked faster on medial-lateral (ON prosthetic leg, 60.98cm;1.13m/s) than rocky (ON prosthetic leg, 57.68cm; 1.06m/s), and rolling hills (ON prosthetic leg, 54.24cm; 0.97m/s). Step width was greater when walking on rocky surfaces (ON prosthetic leg, 15.03cm) than medial-lateral (ON prosthetic leg,14.33cm) and hilly surfaces (ON prosthetic leg, 13.55cm).

### Kinematic gait parameters

**Fig 2:**
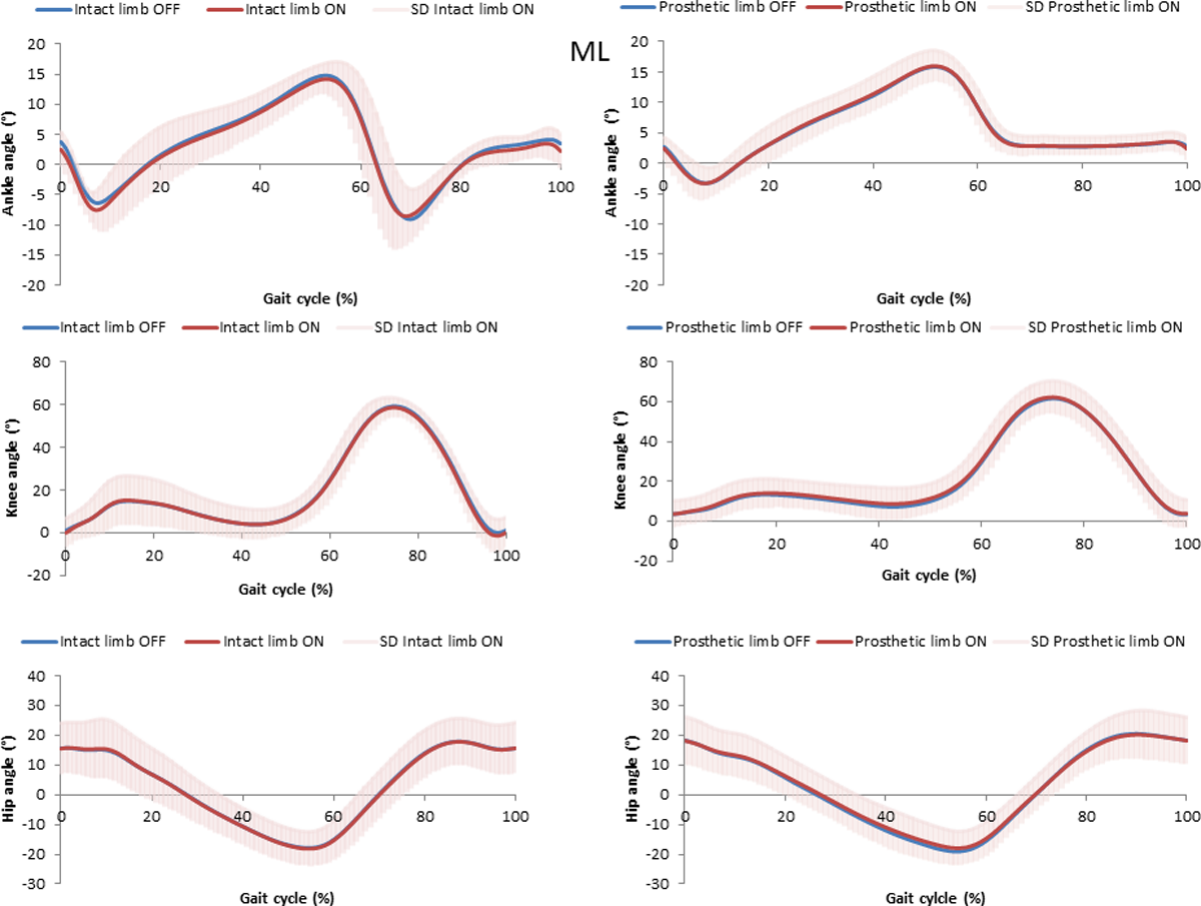
Joints angles for prosthetics and intact limb with the elevated vacuum system ON and OFF for the medial-lateral surface. Average for all participants, standard deviation for ON in gray.

**Fig 3:**
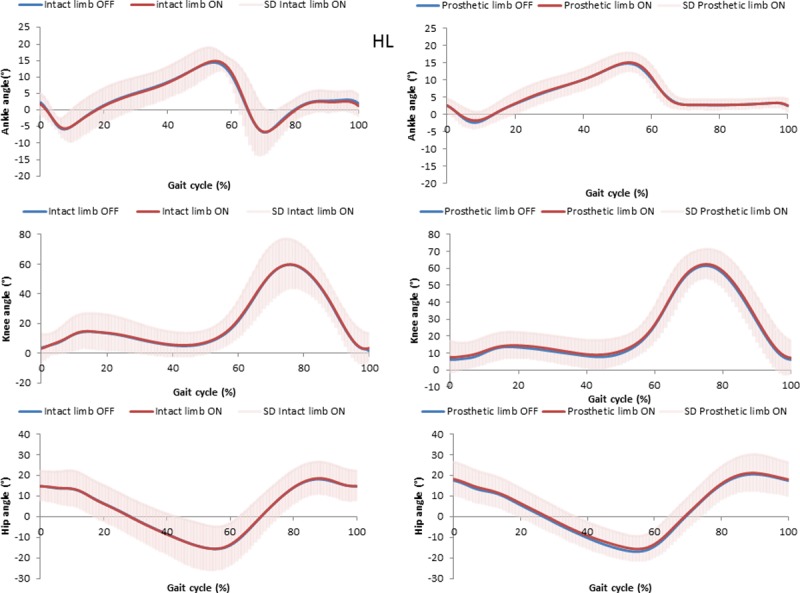
Joints angles for prosthetics and intact limb with the elevated vacuum system ON and OFF for the rolling hills surface. Average for all participants, standard deviation for ON in gray.

**Fig 4:**
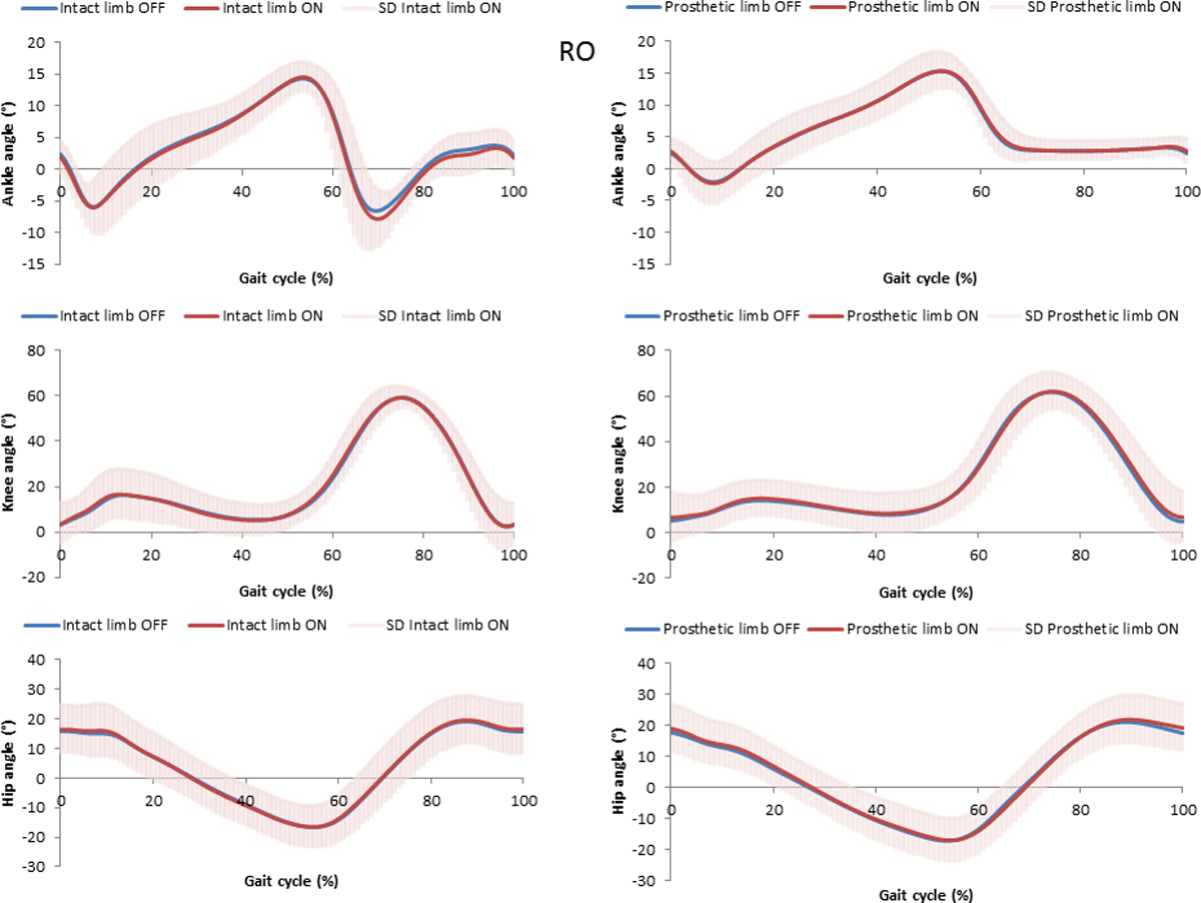
Joints angles for prosthetics and intact limb with the elevated vacuum system ON and OFF for the rocky surface. Average for all participants, standard deviation for ON in gray.

Mean and standard deviation for kinematic parameters are displayed in Table 3 and Figs 2–4. Significant differences were found between vacuum ON and OFF for few parameters, but the differences were very small and may not be clinically significant. For example, a statistically significant difference between vacuum conditions was found for pelvis angle range of motion (rotation) for prosthetic and intact limb, but the difference was less than 2 degree. Compared to medial-lateral translations (ON prosthetic leg, 3.6°), participants increased knee flexion at initial contact for rolling hills (ON prosthetic leg, 7.5°) and rocky surfaces (ON prosthetic leg, 6.7°).

**Table 3:** Mean and standard deviation (in brackets) of kinematic gait parameters with the elevated vacuum system ON and OFF during medial-lateral, rolling hills, and rocky uneven surfaces.

Medial-Lateral								
	Prosthetic Limb				Intact Limb			
	OFF	ON	*p*^*≠*^	*d*^*≠*^	OFF	ON	*p*	*d*
Peak Ankle plantar flexion during early stance	-3.8 (2.6)	-3.9 (2.6)	0.450	0.3	-7.8 (4.8)	-8.5 (4.1)	0.036*	0.5
Peak Ankle dorsiflexion during stance	16.4 (2.7)	16.3 (2.6)	1.000	0.0	15.6 (2.8)	15.3 (2.5)	1.000	0.2
Ankle range of motion	20.2 (3.5)	20.2 (4.0)	0.888	0.2	28.2 (6.1)	28.0 (4.8)	1.000	0.1
Knee flexion at initial contact	3.7 (8.7)	3.6 (6.9)	0.180	0.4	1.3 (8.2)	0.3 (6.9)	0.066	0.4
Peak knee flexion during swing	63.6 (8.6)	63.1 (8.5)	1.000	0.0	60.7 (5.1)	59.8 (4.5)	1.000	0.0
Knee range of motion	62.5 (6.0)	61.6 (6.6)	1.000	0.1	63.4 (6.2)	63.0 (6.5)	1.000	0.0
Peak hip flexion during early stance	18.4 (7.9)	18.3 (8.1)	1.000	0.1	17.0 (7.9)	17.1 (9.3)	1.000	0.1
Peak hip flexion during swing	21.1 (8.0)	20.7 (8.1)	0.600	0.3	18.7 (8.2)	18.5 (7.9)	1.000	0.1
Hip range of motion	40.7 (5.4)	39.3 (5.8)	0.609	0.3	37.8 (3.9)	37.6 (4.8)	1.000	0.1
Pelvis range of motion (sagittal)	3.9 (1.6)	4.1 (1.7)	1.000	0.0	3.8 (1.6)	4.0 (1.6)	1.000	0.0
Pelvis range of motion (frontal)	5.6 (1.9)	5.9 (1.8)	0.036*	0.5	5.6 (1.8)	5.9 (1.7)	0.198	0.4
Pelvis range of motion (rotation)	8.7 (4.2)	8.0 (3.3)	0.099	0.4	8.5 (4.3)	7.6 (3.1)	0.070	0.4
Rolling hills								
	Prosthetic Limb				Intact Limb			
	OFF	ON	*p*	*d*	OFF	ON	p	d
Peak Ankle plantar flexion during early stance	-2.9 (2.0)	-2.5 (2.5)	0.936	0.2	-7.4 (4.3)	-7.1 (4.2)	1.000	0.0
Peak Ankle dorsiflexion during stance	15.6 (3.0)	15.7 (2.9)	1.000	0.0	16.2 (2.4)	16.2 2.3)	1.000	0.0
Ankle range of motion	18.5 (3.4)	18.2 (3.7)	1.000	0.1	27.3 5.4)	26.8 5.1)	1.000	0.2
Knee flexion at initial contact	6.1 (9.1)	7.5 (9.8)	0.765	0.3	3.8 (8.8)	3.2 (9.4)	1.000	0.1
Peak knee flexion during swing	63.0 (9.2)	64.1 (9.8)	0.740	0.5	60.7 (6.3)	61.0 5.8)	1.000	0.2
Knee range of motion	61.0 (6.8)	61.2 (7.4)	1.000	0.0	62.3 (6.1)	62.6 (5.6)	1.000	0.0
Peak hip flexion during early stance	17.8 8.3)	18.4 (8.3)	0.590	0.3	16.6 (8.2)	16.2 (8.6)	1.000	0.1
Peak hip flexion during swing	21.2 (8.6)	21.8 (9.1)	1.000	0.2	19.7 (8.8)	19.4 (9.1)	1.000	0.1
Hip range of motion	39.1 7.9)	38.4 (5.6)	1.000	0.2	37.0 (4.4)	35.7 (3.7)	1.000	0.2
Pelvis range of motion (sagittal)	3.9 (1.9)	3.6 (1. 6)	1.000	0.2	3.6 (1.5)	3.6 (1.6)	1.000	0.2
Pelvis range of motion (frontal)	5.6 (1.5)	5.8 (1.8)	1.000	0.2	5.5 (1.5)	5.7 (1.8)	1.000	0.1
Pelvis range of motion (rotation)	8.4 (4.2)	6.8 (2.5)	0.036*	0.7	8.3 (4.0)	7.0 (2.5)	0.033*	0.7
Rocky								
	Prosthetic Limb				Intact Limb			
	**OFF**	**ON**	*p*	*d*	OFF	ON	*p*	*d*
Peak Ankle plantar flexion during early stance	-3.0 (2.4)	-2.9 (3.3)	1.000	0.2	-6.9 (4.9)	-7.3 (4.8)	0.520	0.3
Peak Ankle dorsiflexion during stance	16.0 (2.9)	16.0 (2.8)	1.000	0.2	15.8 (2.5)	15.6 (2.4)	1.000	0.1
Ankle range of motion	19.0 (3.6)	19.0 (4.3)	0.784	0.3	27.4 (5.8)	27.5 (5.4)	1.000	0.1
Knee flexion at initial contact	5.3 (7.9)	6.7 (11.3)	1.000	0.2	3.3 (8.8)	3.5 (9.5)	1.000	0.1
Peak knee flexion during swing	63.2 (8.5)	63.7 (8.9)	1.000	0.2	60.5 (5.0)	60.7 (4.8)	1.000	0.2
Knee range of motion	61.0 (6.0)	60.8 (6.8)	0.639	0.3	61.7 (8.0)	61.7 (7.9)	1.000	0.0
Peak hip flexion during early stance	17.9 (7.6)	19.2 (7.9)	0.176	0.4	17.1 (8.2)	17.7 (8.7)	1.000	0.0
Peak hip flexion during swing	21.7 (7.9)	22.4 (8.0)	0.630	0.3	19.8 (7.8)	20.2 (8.4)	1.000	0.1
Hip range of motion	39.5 (7.0)	39.9 (5.3)	1.000	0.1	37.6 (3.6)	37.7 (4.5)	1.000	0.4
Pelvis range of motion (sagittal)	3.7 (2.0)	4.0 (1.7)	1.000	0.2	3.5 (1.9)	4.1 (1.6)	0.156	0.4
Pelvis range of motion (frontal)	5.9 (1.3)	5.8 (1.6)	1.000	0.1	6.0 (1.3)	6.0 (1.7)	0.594	0.0
Pelvis range of motion (rotation)	8.3 (4.5)	7.4 (2.8)	0.036*	0.5	8.3 (4.4)	7.3 (2.0)	0.176	0.4

*p* and *d* are between OFF and ON results. * *p*<0.05

## Discussion

This research compared gait with the Unity suspension system to inactive vacuum when walking over various surfaces with continuous perturbations. All results were compared and analysed when vacuum was ON and OFF (prosthetics and intact limb). Small differences between vacuum conditions were found for most temporal-spatial and kinematic gait parameters, but differences correspond to small effect sizes and were not considered clinically significant. Therefore, the hypothesis that temporal-spatial and kinematic gait parameters would be different between elevated vacuum and inactive vacuum conditions was not supported.

### Temporal-spatial gait parameters

Participants increased step length and walked faster on the medial-lateral surface than rocky, and rolling hills, and for both vacuum conditions. These gait changes were consistent with the literature, where transtibial amputees and able-bodied individuals used adaptive gait strategies to avoid the loss of balance in a stability-challenging environment [24].

Previous research showed that elevated vacuum suspension can improve socket fit, which improved gait, balance, and satisfaction [25]. In our study, step time and stance time were symmetric between prosthetic and intact limbs, for both vacuum conditions, during walking with continuous perturbations. However, the symmetry index was higher than 10 percent for step length. As reported in the literature, transtibial amputees can develop step length asymmetry on non-level surfaces [12]. Prosthetic step length and single limb support time were slightly smaller than intact limb results, for both vacuum conditions, and can be explain by amputees relying more on their intact leg then prosthetic leg [10]. Participants may have tried to compensate for gait imbalances with their intact leg, to provide better stability during prosthetic single limb support [12].

### Kinematic gait parameters

Prosthetic limb ankle, knee, hip, and pelvis range of motion differences between ON and OFF were very small. Similarly, Xu et al., and Ferreira reported no significant differences between vacuum conditions for other elevated vacuum systems during level walking [8,10].

Compared to medial-lateral translations, participants increased knee flexion at initial foot contact for rolling hills and rocky surfaces. Increased knee flexion can contribute to an overall lowering of the body to increase stability [13,14]. This could also contribute to the smaller step length for rolling hills and rocky surfaces observed in this study. These findings suggest that rolling hills was the most challenging surface as participants increased knee flexion at initial contact and walked with shorter steps for rolling hills compared to the other non-level surfaces.

The protocol in this study tested the immediate effects of the inactive vacuum condition since the vacuum was only inactive during the data collection session. If vacuum was inactive for a longer period, socket fit may worsen as limb volume changes, thereby leading to greater gait differences or discomfort. Daily volume loss is a concern for good prosthetic fit. Elevated vacuum systems have shown to prevent volume loss or even increase limb volume (3.5%) [2]. Therefore, minimizing limb volume changes may be especially important for maximizing gait stability on non-level terrain for transtibial amputees. Future research could examine long-term effects of an inactive vacuum system while walking on non-level surfaces to better understand how volume changes affect gait performance during more challenging walking scenarios. This research investigated a high functioning group with transtibial amputation (K3, K4). Future research should examine effects of the Unity system’s on gait performance and comfort for individuals with a transtibial amputation at a lower activity level. Additionally, gait biomechanics have primarily focused on differences in the lower body and future work should explore effects of elevated vacuum system on upper body kinematics for non-level walking.

## Conclusion

This study examined transtibial amputee gait when walking with the Össur Unity elevated vacuum system in active and inactive vacuum conditions, for three continuous perturbation walking surfaces. Significant differences were found between vacuum ON and OFF for few gait parameters, but the differences were small and were considered not clinically significant. Therefore, gait performance in a high functioning amputee population would not be immediately affected following a mechanical vacuum pump failure. However, if the vacuum were off for an extended period the residual limb volume would be expected to fluctuate, resulting in inferior socket fit [5]. Further research on elevated vacuum effects on amputee comfort would be beneficial to assist in clinical decision-making.

## Supporting information

S1 Appendix Appendix A (six degree of freedom 57 marker set). (DOCX)
