# Supplementary material for: Effects of the unity vacuum suspension system on transtibial gait for simulated non-level surfaces
Source: PLoS One. 2018 Jun 14;13(6):e0199181. doi: 10.1371/journal.pone.0199181 (PMC6002056; doi:10.1371/journal.pone.0199181)
Supplement: S1 Appendix — (DOCX) [file pone.0199181.s001.docx]

# Appendix A (six degree of freedom 57 marker set)

|  | **Marker Label** | **Description** |  | **Marker Label** | **Description** | |
| --- | --- | --- | --- | --- | --- | --- |
|  | **Head** |  |  | **Left Thigh** | | |
| **1** | RFHD | Right forehead | **34** | LTH1 | Left Thigh Plate (anterior, superior) | |
| **2** | LFHD | Left forehead | **35** | LTH2 | Left Thigh Plate (anterior, inferior) | |
| **3** | RBHD | Right back head | **36** | LTH3 | Left Thigh Plate (posterior, inferior) | |
| **4** | LBHD | Left back head | **37** | LTH4 | Left Thigh Plate (posterior, superior) | |
|  | **Trunk** |  |  | **Left Shank** | | |
| **5** | C7 | 7th Cervical Vertebra | **38** | LSK1 | Left Shank Plate (anterior, superior) | |
| **6** | T8 | 8th Thoracic Vertebra (or a stable area on the harness) | **39** | LSK2 | Left Shank Plate (anterior, inferior) | |
| **7** | RBAC | Right back marker (or a stable area on the harness near the right shoulder) | **40** | LSK3 | Left Shank Plate (posterior, inferior) | |
| **8** | STRN | Sternal Notch | **41** | LSK4 | Left Shank Plate (posterior, superior) | |
| **9** | XYPH | Xyphoid Process |  |  |  | |
|  | **Left Arm** | |  | **Left Foot** | | |
| **10** | LSHO | Left acromion | **42** | LHEE | Left heel | |
| **11** | LUA1 | Left upper arm proximal and posterior | **43** | LLHL | Left lateral heel | |
| **12** | LUA2 | Left upper arm anterior | **44** | L5MT | Left 5th Metatarsal Head | |
| **13** | LUA3 | Left upper arm distal and posterior | **45** | LTOE | Left 2nd Metatarsal Head | |
| **14** | LFAL | Left forearm lateral |  | **Right Thigh** | | |
| **15** | LFAM | Left forearm medial | **46** | RTH1 | Right Thigh Plate (anterior, superior) | |
| **16** | LWRR | Left radial styloid | **47** | RTH2 | Right Thigh Plate (anterior, inferior) | |
| **17** | LWRU | Left ulnar styloid | **48** | RTH3 | Right Thigh Plate (posterior, inferior) | |
| **18** | LFIN | Left hand | **49** | RTH4 | Right Thigh Plate (posterior, superior) | |
|  | **Right Arm** | |  | **Right Shank** | | |
| **19** | RSHO | Right acromion | **50** | RSK1 | Right Shank Plate (anterior, superior) | |
| **20** | RUA1 | Right upper arm proximal and posterior | **51** | RSK2 | Right Shank Plate (anterior, inferior) | |
| **21** | RUA2 | Right upper arm anterior | **52** | RSK3 | Right Shank Plate (posterior, inferior) | |
| **22** | RUA3 | Right upper arm distal and posterior | **53** | RSK4 | Right Shank Plate (posterior, superior) | |
| **23** | RFAL | Right forearm lateral |  | **Right Foot** | |  |
| **24** | RFAM | Right forearm medial | **54** | RHEE | Right heel | |
| **25** | RWRR | Right radial styloid | **55** | RLHL | Right lateral heel | |
| **26** | RWRU | Right ulnar styloid | **56** | R5MT | Right 5th Metatarsal Head | |
| **27** | RFIN | Right hand | **57** | RTOE | Right 2nd Metatarsal Head | |
|  | **Pelvis** |  |  |  |  | |
| **28** | RASI | Right Anterior Superior Iliac Spine |  |  |  | |
| **29** | RASI2 | Right Iliac Crest |  |  |  | |
| **30** | LASI | Left Anterior Superior Iliac Spine |  |  |  | |
| **31** | LASI2 | Left Iliac Crest |  |  |  | |
| **32** | RPSI | Right Posterior Superior Iliac Spine |  |  |  | |
| **33** | LPSI | Left Posterior Superior Iliac Spine |  |  |  | |

**Landmarks**

Virtual markers (digitizing landmarks) were used to define segment ends.

Order of digitizing landmarks:

|  | **Marker Label** | **Reference markers** | **Description** |
| --- | --- | --- | --- |
| **1** | _LANL | LSK1, LSK2, LSK3 | Left Lateral Malleolus |
| **2** | _LANM | LSK1, LSK2, LSK3 | Left Medial Malleolus |
| **3** | _LKNL | LSK1, LSK2, LSK3 | Left Lateral Femoral Condyle |
| **4** | _LKNM | LSK1, LSK2, LSK3 | Left Medial Femoral Condyle |
| **5** | _LGTR | LTH1, LTH2, LTH3 | Left Greater Trochanter |
| **6** | _LILL | RASI, RPSI, LPSI | Left Iliac Crest |
| **7** | _RANL | RSK1, RSK2, RSK3 | Right Lateral Malleolus |
| **8** | _RANM | RSK1, RSK2, RSK3 | Right Medial Malleolus |
| **9** | _RKNL | RSK1, RSK2, RSK3 | Right Lateral Femoral Condyle |
| **10** | _RKNM | RSK1, RSK2, RSK3 | Right Medial Femoral Condyle |
| **11** | _RGTR | RTH1, RTH2, RTH3 | Right Greater Trochanter |
| **12** | _RILL | LASI, LPSI, RPSI | Right Iliac Crest |
| **13** | _LSHA | C7, STRN, XYPH | Left Shoulder Joint Center - Anterior |
| **14** | _LSHP | C7, STRN, XYPH | Left Shoulder Joint Center - Posterior |
| **15** | _LELL | LUA1, LUA2, LUA3 | Left Lateral Epicondyle |
| **16** | _LELM | LUA1, LUA2, LUA3 | Left Medial Epicondyle |
| **17** | _RSHA | C7, STRN, XYPH | Right Shoulder Joint Center - Anterior |
| **18** | _RSHP | C7, STRN, XYPH | Right Shoulder Joint Center - Posterior |
| **19** | _RELL | RUA1, RUA2, RUA3 | Right Lateral Epicondyle |
| **20** | _RELM | RUA1, RUA2, RUA3 | Right Medial Epicondyle |
